# A New in Vitro Model of GDLD by Knocking Out *TACSTD2* and Its Paralogous Gene *EpCAM* in Human Corneal Epithelial Cells

**DOI:** 10.1167/tvst.7.6.30

**Published:** 2018-12-21

**Authors:** Peng Xu, Chifune Kai, Satoshi Kawasaki, Yuki Kobayashi, Kouji Yamamoto, Motokazu Tsujikawa, Ryuhei Hayashi, Kohji Nishida

**Affiliations:** 1 Department of Ophthalmology, Osaka University Graduate School of Medicine, Osaka, Japan; 2 Department of Ocular Immunology and Regenerative Medicine, Osaka University Graduate School of Medicine, Osaka, Japan; 3 Faculty of Medicine, Osaka University Graduate School of Medicine, Osaka, Japan; 4 Department of Biostatistics, Yokohama City University, School of Medicine, Kanagawa, Japan; 5 Division of Health Sciences, Osaka University Graduate School of Medicine, Osaka, Japan; 6 Department of Stem Cells and Applied Medicine, Osaka University Graduate School of Medicine, Osaka, Japan

**Keywords:** gelatinous drop-like corneal dystrophy, TACSTD2, EpCAM, claudin, tight junction

## Abstract

**Purpose:**

Gelatinous drop-like corneal dystrophy (GDLD) is a rare autosomal recessive corneal dystrophy that causes severe vision loss. Because of its poor prognosis, there is a demand for novel treatments for GDLD. Here, we establish a new in vitro disease model of GDLD based on immortalized human corneal epithelial (HCE-T) cells.

**Methods:**

By using transcription activator-like effector nuclease plasmids, tumor-associated calcium signal transducer 2 (*TACSTD2*) and its paralogous gene, epithelial cell adhesion molecule (*EpCAM*), were knocked out in HCE-T cells. Fluorescence-activated cell sorting was performed to obtain cells in which both *TACSTD2* and *EpCAM* were knocked out (DKO cells). In DKO cells, the expression levels and subcellular localizations of claudin (CLDN) 1, 4, and 7, and ZO-1 were investigated, along with epithelial barrier function. By using DKO cells, the feasibility of gene therapy for GDLD was also investigated.

**Results:**

DKO cells exhibited decreased expression and aberrant subcellular localization of CLDN1 and CLDN7 proteins, as well as decreased epithelial barrier function. Transduction of the *TACSTD2* gene into DKO cells nearly normalized expression levels and subcellular localization of CLDN1 and CLDN7 proteins, while significantly increasing epithelial barrier function.

**Conclusions:**

We established an in vitro disease model of GDLD by knocking out *TACSTD2* and its paralogous gene, *EpCAM,* in HCE-T cells. This cell line accurately reflected pathological aspects of GDLD.

**Translational Relevance:**

We expect that the cell line will be useful to elucidate the pathogenesis of GDLD and develop novel treatments for GDLD.

## Introduction

Gelatinous drop-like corneal dystrophy (GDLD; OMIM: 204870), which was first reported in 1914 by Nakaizumi,^1^ is a rare autosomal recessive inheritable corneal dystrophy.^2,3^ Clinical manifestations of GDLD include visual impairment, photophobia, irritation, redness, and tearing, which occur from as early as the first decade of life.^2,3^ Histologically, amyloid depositions are observed at the subepithelial region of the cornea.^4^ These amyloid depositions gradually increase in number and size, severely impairing vision. The epithelial barrier function of corneal epithelium is severely reduced in GDLD.^5,6^ If vision decreases, lamellar or penetrating keratoplasty is typically performed for the patient.^2,3^ However, amyloid depositions often relapse in the transplanted cornea within several years after operation,^2^ and repetitive keratoplasties must often be performed to restore vision.^7,8^ In certain severe cases, keratoprosthesis is considered.^9–11^ There is a compelling demand to develop novel treatment methods for GDLD.

The gene encoding tumor-associated calcium signal transducer 2 (*TACSTD2*) has been confirmed to be responsible for the onset of GDLD.^12,13^ The TACSTD2 protein is a 35- to 49-kDa transmembrane glycoprotein,^14,15^ which was first identified as a cell surface marker for trophoblast cells.^16^ TACSTD2 was reported to bind to the tight junction-related proteins, claudin 1 (CLDN1) and CLDN7, preventing them from degradation by the proteasome.^17^ In the corneal epithelial cells of GDLD patients, CLDN1 and CLDN7 exhibited decreased expression and altered subcellular localization from cell membrane to cytoplasm,^17,18^ which may explain the clinically observed reduction in epithelial barrier function. The reduced epithelial barrier function may allow permeation of tear fluid into corneal tissue. Lactoferrin, a major component of tear proteins,^19^ is thought to be the major component of subepithelial amyloid deposition,^20^ possibly through its amyloidogenic tendency.^21,22^ However, the detailed pathogenesis of GDLD remains unclear.

In the present study, we established a cell line to be used as an in vitro disease model of GDLD by knocking out *TACSTD2* and its paralogous gene, epithelial cell adhesion molecule (*EpCAM*)*,* in immortalized human corneal epithelial (HCE-T) cells. This cell line demonstrated markedly reduced epithelial barrier function with decreased expression and altered subcellular localization of CLDN1 and CLDN7 proteins, consistent with pathological changes found in the corneal epithelial cells of GDLD. We expect that this cell line will be useful for further elucidation of the pathogenesis of GDLD, as well as for the development of novel treatment methods for GDLD.

## Methods

### Ethical Approval

The present study followed the tenets of the Declaration of Helsinki. Written informed consent was obtained from patients after explanation of the nature and possible consequences of this study. All experimental procedures in the present study were performed under the approval of the institutional review board for human study and the Gene Modification Experiments Safety Committee of Osaka University.

### Antibodies

All antibodies used in this study are listed in Table 1.

**Table 1:**
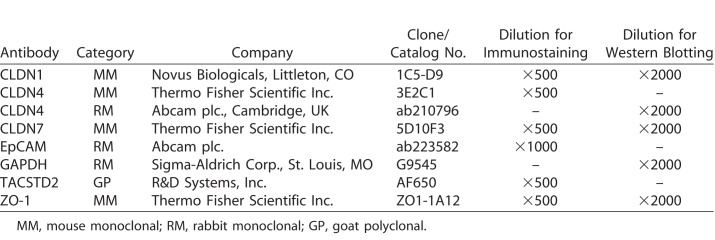
List of Antibodies Used in This Study

### Oligomers

All oligomers used in this study were synthesized by Fasmac Co., Ltd. (Atsugi, Japan) (Table 2).

**Table 2:**
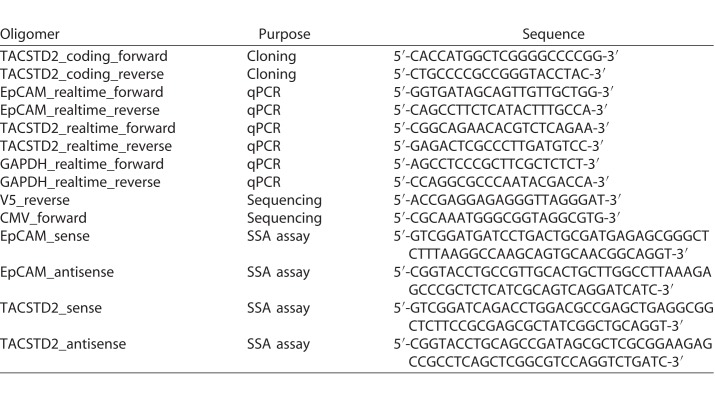
List of Oligomers Used in This Study

### Human Corneal Tissues

Normal human corneal tissues were obtained from an eye bank (SightLife, Seattle, WA). Cryosections and an RNA sample were obtained from the tissue. GDLD corneal tissue was obtained from a GDLD patient at surgery.

### Cell Culture

HCE-T cells (RCB2280), the most commonly used immortalized human corneal epithelial cells, were obtained from a cell bank (RIKEN BioResource Center, Tsukuba, Japan). The cells were cultured in a supplemented hormonal epithelial medium (SHEM), which contains Dulbecco's modified Eagle medium (DMEM)/F-12 (1:1) (Nacalai Tesque Inc., Kyoto, Japan), 10% fetal bovine serum (FBS), 0.5X Insulin-Transferrin-Selenium-Ethanolamine Solution (Thermo Fisher Scientific, Inc., Waltham, MA), and 10 ng/mL epidermal growth factor (R&D Systems, Inc., Minneapolis, MN). Also obtained from the RIKEN cell bank were 293T cells (RCB2202). The cells were cultured in DMEM (Nacalai Tesque Inc.), supplemented with 10% FBS.

At the cell bank, these cells had been tested for various biological aspects, including mycoplasma infection, cell viability, and morphology. Short tandem repeat polymorphism analysis had also been performed to guarantee cell origin and lack of cross contamination.

### Immortalization of Corneal Epithelial Cells

Corneal epithelial cells were cultured from GDLD and normal corneal tissues. These cells were cultured in a serum-free medium (CnT-Prime Epithelial Culture Medium; CELLnTEC Advanced Cell Systems AG, Bern, Switzerland) and immortalized as previously reported.^18^

### Subcloning of HCE-T Cells

Subcloning of HCE-T cells was performed by a limited dilution method. Cells were seeded at a density of two cells per well in 96-well plates. Cells that grew in wells with a single initial colony were chosen for subsequent culture.

### Gene Knockout by Transcription Activator-Like Effector Nuclease (TALEN)

TALEN target sequences were designed by an on-line tool, TALEN Targeter (https://tale-nt.cac.cornell.edu/node/add/talen-old; available in the public domain). TALEN plasmids were constructed in accordance with the Platinum Gate TALEN construction protocol 2014, version 1.0 (https://media.addgene.org/cms/files/Platinum_Gate_protocol.pdf; available in the public domain). Constructed plasmids were validated by restriction enzyme digestion, and their cutting efficiency was confirmed by single-strand annealing (SSA) assay.^23^ For positive control experiment, TALEN expression plasmids (HPRT1_B TALEN-R and HPRT1_B TALEN-L) were used. For super-positive control experiment, TALEN expression plasmids (HPRT1_B TALEN-NC-R and HPRT1_B TALEN-NC-L) were used. For negative control experiment, TALEN expression plasmids for *TACSTD2* gene were used. For these control experiments, an SSA reporter plasmid (pGL4-SSA-HPRT1) was used to report their cutting efficiency.

HCE-T cells were seeded in a 12-well plate at a density of 180,000 cells/well. Twelve hours later, TALEN plasmids (1 μg) were transfected into the HCE-T cells by using a commercial transfection reagent (3.5 μL, FuGENE HD Transfection Reagent; Promega Corporation, Madison, WI).

### Fluorescence-Activated Cell Sorting (FACS)

Cells were trypsinized and blocked in a FACS buffer containing 2% FBS diluted in Dulbecco's phosphate-buffered saline (D-PBS(-); Wako Pure Chemical Industries, Ltd., Osaka, Japan). The cells were incubated with a primary antibody diluted in FACS buffer at 4°C for 30 minutes. After they were washed with FACS buffer, the cells were incubated with secondary antibodies (Alexa Fluor 568 Donkey anti-Rabbit IgG, Alexa Fluor 647 Donkey anti-Goat IgG, 1:200 dilution; Thermo Fisher Scientific, Inc.) diluted in FACS buffer at 4°C for 30 minutes. After they were washed with FACS buffer, the cells were suspended in FACS buffer and subjected to FACS analysis by using a commercial cell sorter (SH800S; Sony Biotechnology, San Jose, CA).

### Quantitative Polymerase Chain Reaction (qPCR)

RNA was reverse transcribed by using a commercial kit (ReverTra Ace qPCR RT Master Mix with gDNA Remover; TOYOBO Co., Ltd., Osaka, Japan). The cDNA was amplified with a primer pair (3 pmol) in a reaction mixture (10 μL, SYBR Premix DimerEraser; Takara Bio Inc., Kusatsu, Japan) by using a commercial PCR machine (7500 Fast Real-Time PCR system; Thermo Fisher Scientific, Inc.). Thermal cycling conditions were 40 cycles of denaturation at 95°C for 3 seconds and annealing/elongation at 60°C for 30 seconds. All experiments were performed in duplicate.

### Immunostaining

Cells were grown on a collagen-coated (Cellmatrix Type I-P; Nitta Gelatin Inc., Osaka, Japan) commercial culture glass slide (Nunc Lab-Tek Chamber Slide System; Thermo Fisher Scientific, Inc.). The cells were fixed with 4% paraformaldehyde (Nacalai Tesque Inc.) at room temperature for 20 minutes or with 95% ethanol at 4°C for 30 minutes, permeabilized in a solution containing 0.1% Triton X-100 diluted in D-PBS(-) at room temperature for 20 minutes. The cells were blocked in a blocking buffer containing 5% donkey serum and 0.3% Triton X-100 diluted in Tris-buffered saline (TBS; Takara Bio, Inc.) at room temperature for 1 hour. The cells were incubated with primary antibody diluted in the blocking buffer at 4°C overnight. After they were washed with 0.05% Triton X-100 diluted in D-PBS(-) (DPBS-T), the cells were incubated with secondary antibodies (Alexa Fluor 488 Donkey anti-Mouse IgG, Alexa Fluor 594 Donkey anti-Rabbit IgG, Alexa Fluor 647 Donkey anti-Goat IgG, 1:1000 dilution; Thermo Fisher Scientific, Inc.), diluted in the blocking buffer in the presence of 1 ng/mL Hoechst 33342 dye (Sigma-Aldrich Co., Darmstadt, Germany), at room temperature for 1 hour. For some experiments, 5 units/mL phalloidin (Alexa Fluor 488 phalloidin; Thermo Fisher Scientific, Inc.) was used to stain cell-cell borders. After they were washed with DPBS-T, the cells were mounted with a commercial mounting medium (PermaFluor Aqueous Mounting Medium; Thermo Fisher Scientific, Inc.), covered with a glass coverslip, and photographed by using a commercial confocal laser scanning microscope (LSM 710; Carl Zeiss AG, Jena, Germany).

### Western Blotting Analysis

Cells were lysed with radioimmunoprecipitation assay buffer (Nacalai Tesque Inc.) containing 1X protease inhibitor mixture (Protease Inhibitor Cocktail for Use With Mammalian Cell and Tissue Extracts; Nacalai Tesque Inc.) and incubated at 4°C for 30 minutes. After brief centrifugation, supernatant of the lysate was mixed with 0.2 volume of 6X Laemmli sample buffer containing 300 mM Tris-HCl (pH 6.8), 60% glycerol, 12% sodium dodecyl sulfate, 0.03% bromophenol blue, and 300 mM dithiothreitol, and denatured at 70°C for 10 minutes. The lysate was electrophoresed on a commercial polyacrylamide gel (4%–20% Mini-PROTEAN or Criterion TGX Precast Gel; Bio-Rad Laboratories, Inc., Hercules, CA) and transferred to a commercial polyvinylidene fluoride membrane (Trans-Blot Turbo Transfer System Transfer Pack; Bio-Rad Laboratories, Inc.). The membrane was washed in the TBS containing 0.1% Tween-20 (TBS-T) and blocked in a blocking buffer (Blocking One; Nacalai Tesque Inc.) at room temperature for 1 hour. The membrane was incubated with a primary antibody diluted in the blocking buffer at 4°C overnight. After the membrane was washed with TBS-T, it was incubated with a secondary antibody (anti-rabbit IgG, HRP-linked antibody, or anti-mouse IgG, HRP-linked antibody; Cell Signaling Technology, Inc., Danvers, MA) diluted in the blocking buffer at room temperature for 1 hour. After the membrane was washed with TBS-T, a commercial chemiluminescent reagent (ECL Advance Western Blotting Detection Kit; GE Healthcare, Little Chalfont, UK) was applied to the membrane. The chemiluminescent signal was detected by using a commercial intelligent dark box (ChemiDoc XRS+ System; Bio-Rad Laboratories, Inc.). All experiments were done in hexaplicate.

### Lentivirus Production and Transduction

The coding sequence of the *TACSTD2* gene was amplified by PCR. The PCR product was cloned into an entry vector (pENTR/D-TOPO; Thermo Fisher Scientific, Inc.). The cloned insert was transferred to a destination vector (pLenti6.3/V5–DEST Gateway Vector; Thermo Fisher Scientific, Inc.) by using a commercial enzyme for in vitro recombination (Gateway LR Clonase II Enzyme mix; Thermo Fisher Scientific, Inc.). The lentiviral vector was transfected into 293T cells, along with packaging plasmids (ViraPower Lentiviral Packaging Mix; Thermo Fisher Scientific, Inc.), by using a commercial transfection reagent (TransIT-293 Transfection Reagent; Mirus Bio LLC, Madison, WI). Two days after transfection, the culture medium containing lentivirus particles was collected and briefly centrifuged. Lentivirus transduction was performed in the presence of 5 μg/mL polybrene (Nacalai Tesque Inc.).

### Measurements of Transepithelial Resistance (TER)

Cells were cultured on a 24-well Transwell plate (0.4-μm pore; Falcon Cell Culture Inserts, Corning, Inc., Corning, NY). Resistance between upper and lower chambers of the Transwell plate was measured by using a volt-ohm meter (EVOM II; World Precision Instruments, Sarasota, FL). Background resistance derived from the Transwell plate was subtracted from the obtained resistance data. TER was calculated by multiplying the measured resistance (ohm) by the growth area of the Transwell plate (0.3 cm^2^). All measurements were performed in triplicate.

### Statistical Analysis

Student's *t*-test and 1-way analysis of variance (ANOVA) were carried out using free software (R; The R Foundation, Vienna, Austria).

## Results

### Subcloning of HCE-T Cells

HCE-T cells have been reported to comprise two different cell types: one cell type demonstrates clear cell-cell borders with high epithelial barrier function, whereas the other type demonstrates unclear cell-cell borders with low epithelial barrier function.^24^ We suspected that we could test the effectiveness of a newly developed treatment for GDLD with our model cells; thus, we could plan to assess effectiveness by the difference in epithelial barrier function, with or without the tested treatment. If our model cells were established from cells with low epithelial barrier function, the difference in epithelial barrier function with or without the tested treatment might be small; thus, sensitivity to identify the effectiveness of the tested treatment would be low. Therefore, we performed subcloning of HCE-T cells to obtain cells with high epithelial barrier function.

**Figure 1:**
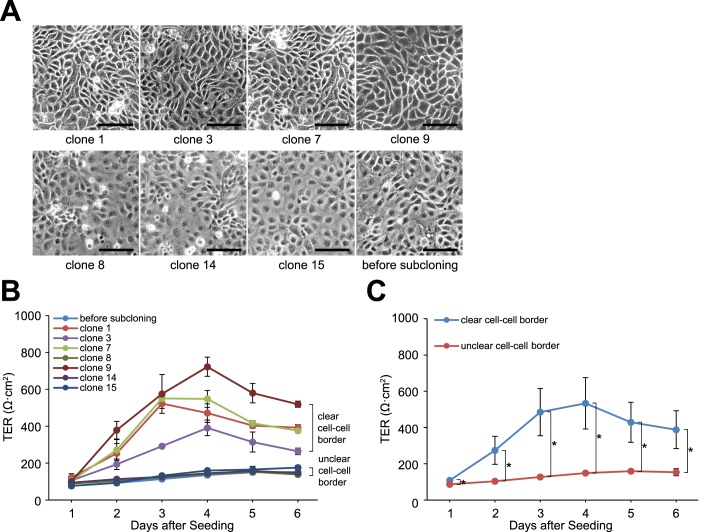
Subcloning of HCE-T cells. (A) Phase contrast images of HCE-T cells before and after subcloning. Scale bars: 50 μm. Note that some HCE-T subclones (clones 1, 3, 7, and 9) demonstrate clear cell-cell borders; other HCE-T subclones (clones 8, 14, and 15), and HCE-T cells before subcloning, demonstrate unclear cell-cell borders. (B) Epithelial barrier function was assessed for HCE-T subclones by measuring TER. Error bars: standard deviations of measurements. (C) Difference in epithelial barrier function between subclones with clear cell-cell borders (HCE-T clones 1, 3, 7, and 9) and those with unclear cell-cell borders (HCE-T clones 8, 14, and 15). Error bars: standard deviations among subclones of each group. Asterisks indicate statistical significance at P < 0.05 (Student's t-test).

Similar to the previous report, we found that some subclones of HCE-T cells demonstrated clear cell-cell borders with high epithelial barrier function, while some subclones demonstrated unclear cell-cell borders with low epithelial barrier function (Fig. 1A, 1B, 1C). It is of interest that populations of HCE-T cells before subcloning demonstrated low epithelial barrier function. We chose clone 9 of the HCE-T cells (HCE-T-9 cells) for subsequent experiments.

### Knockout of *TACSTD2* in HCE-T Cells

**Figure 2:**
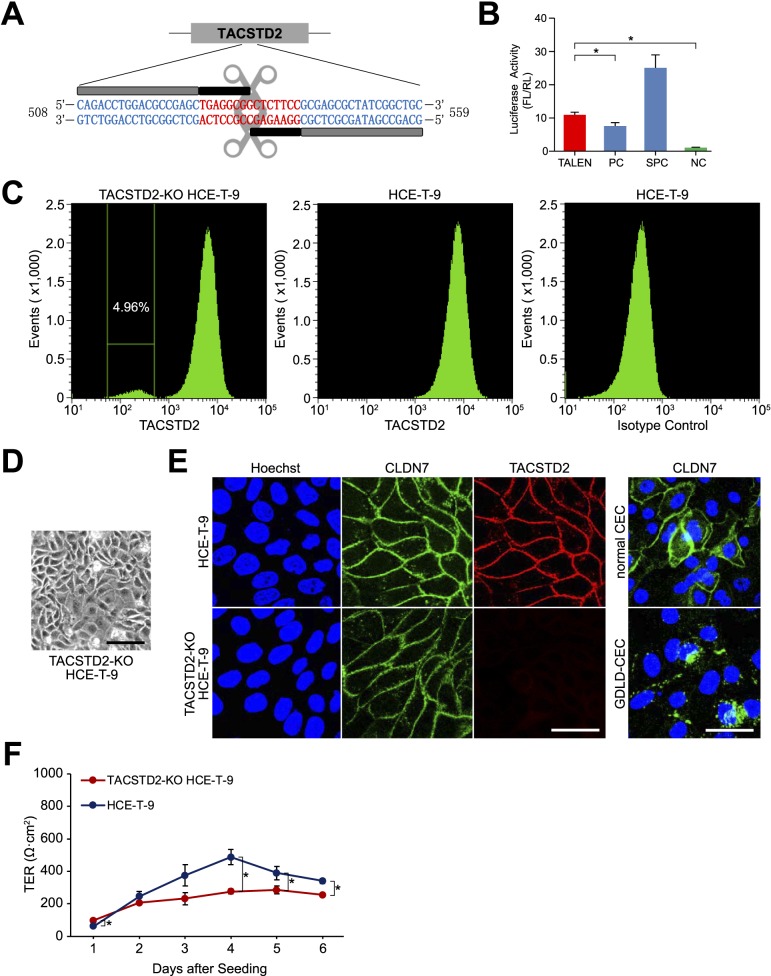
The process of knocking out TACSTD2 in HCE-T clone 9 (HCE-T-9) cells. (A) TALEN target region in the TACSTD2 gene. Sequences of repeat variable diresidue (RVD) and spacer regions are respectively colored in blue and red. (B) Results of SSA assay. Vertical axis denotes the ratio of chemiluminescent signal from firefly luciferase (FL) to the signal from renilla luciferase (RL). PC, positive control; SPC, super-positive control; NC, negative control. Error bars: standard deviations of measurements. Asterisks indicate statistical significance at P < 0.05 (Student's t-test). (C) Results of FACS for HCE-T-9 cells in which TACSTD2 was knocked out (TACSTD2-KO HCE-T-9): cells stained with anti-TACSTD2 antibody (left), cells stained with anti-TACSTD2 antibody (middle), and cells stained with isotype control (right). (D) Phase contrast image of TACSTD2-KO HCE-T-9 cells. Scale bar: 50 μm. (E) Results of immunostaining analysis for CLDN7 and TACSTD2 in HCE-T-9 cells and TACSTD2-KO HCE-T-9 cells. For comparison, included are results of immunostaining analysis for CLDN7 in immortalized corneal epithelial cells derived from a normal cornea (normal CEC) and a GDLD cornea (GDLD-CEC). Scale bars: 50 μm. (F) Results of TER measurements in HCE-T-9 cells and TACSTD2-KO HCE-T-9 cells. Error bars: standard deviations of measurements. Asterisks indicate statistical significance at P < 0.05 (Student's t-test).

We successfully constructed TALEN plasmids for *TACSTD2* (Fig. 2A). The cutting efficiency of the TALEN plasmids was significantly higher than the negative control and even higher than positive control, as confirmed by SSA assay (Fig. 2B). The TALEN plasmids were transfected into HCE-T-9 cells. After 7 days, FACS analysis revealed that approximately 5% of transfected cells were devoid of *TACSTD2* gene expression (Fig. 2C). These *TACSTD2-*negative HCE-T-9 cells (TACSTD2-KO HCE-T-9) (Fig. 2D) were collected for subsequent analyses.

Immunostaining analysis confirmed that TACSTD2-KO HCE-T-9 cells did not express TACSTD2 protein (Fig. 2E). The TER of TACSTD2-KO HCE-T-9 cells was significantly decreased, compared with HCE-T-9 cells (Fig. 2F). However, CLDN7 protein continued to be localized on the cell membrane (Fig. 2E). In GDLD corneal epithelial cells, the subcellular localization of CLDN7 protein was altered from cell membrane to cytoplasm (Fig. 2E). The lack of change in the subcellular localization of CLDN7 protein in the TACSTD2-KO HCE-T-9 cells was quite unexpected.

### Expression of *EpCAM* Gene in HCE-T Cells

**Figure 3:**
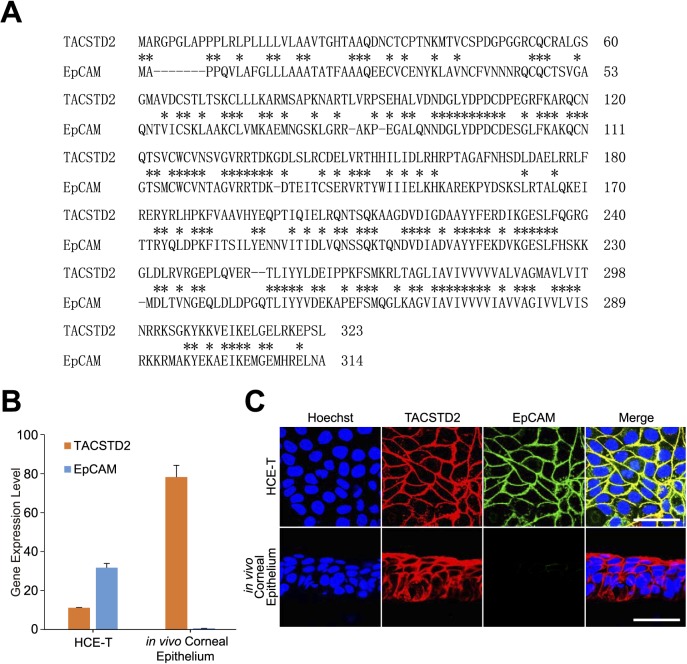
Sequence similarity between TACSTD2 and EpCAM proteins and their expression in HCE-T cells and in vivo human corneal epithelium. (A) Alignment of human TACSTD2 (NP_002353.2) and EpCAM (NP_002354.2) protein sequences. Asterisks indicate identical amino acid pairs and hyphens (-) indicate gaps. The two proteins demonstrate approximately 50% amino acid sequence similarity. (B) Results of qPCR analysis for TACSTD2 and EpCAM genes in HCE-T cells and in vivo human corneal epithelium. Vertical scale denotes copy numbers of indicated genes normalized by copy number of GAPDH gene. Error bars: standard deviations of measurements. (C) Results of immunostaining analysis for TACSTD2 and EpCAM in HCE-T cells and in vivo human corneal epithelium. Scale bars: 50 μm. Note that EpCAM was expressed in HCE-T cells but not in in vivo human corneal epithelium.

*EpCAM* was found to be the most paralogous gene to *TACSTD2*, with an approximate 50% similarity in amino acid sequence (Fig. 3A). A previous study demonstrated that EpCAM protein interacts with CLDN1 and CLDN7 proteins and plays an important role in the formation of tight junctions, similar to TACSTD2 protein.^25^ Thus, we suspected that EpCAM protein may compensate for the loss of TACSTD2 function in TACSTD2-KO HCE-T-9 cells.

In our present study, *EpCAM* was expressed in HCE-T cells, both at mRNA (Fig. 3B) and protein levels (Fig. 3C). In contrast, in vivo human corneal epithelium demonstrated virtually no expression of EpCAM (Fig. 3B, 3C).

### Knockout of *TACSTD2* and *EpCAM* Genes in HCE-T Cells

**Figure 4:**
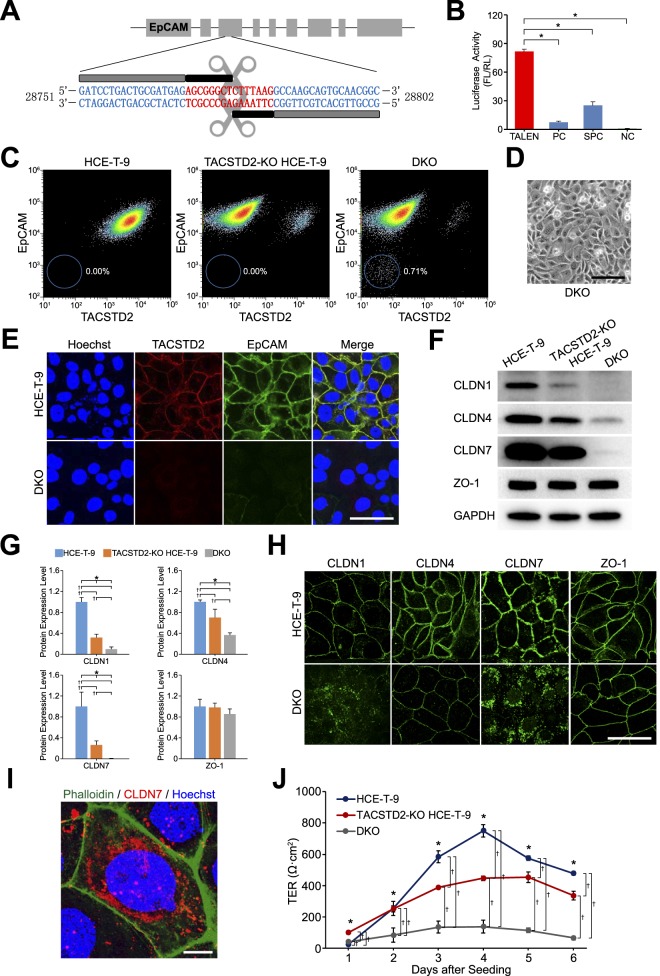
The process of knocking out EpCAM in TACSTD2 knockout (TACSTD2-KO) HCE-T-9 cells. (A) TALEN target region in the EpCAM gene. Sequences of RVD and spacer regions are respectively colored in blue and red. (B) Results of SSA assay. Vertical axis denotes the ratio of chemiluminescent signal from FL to the signal from RL. PC, positive control; SPC, super-positive control; NC, negative control. Error bars: standard deviations of measurements. Asterisks indicate statistical significance at P < 0.05 (Student's t-test). (C) Results of FACS analysis for HCE-T-9 cells (left), TACSTD2-KO HCE-T-9 cells (middle), and HCE-T-9 cells where both TACSTD2 and EpCAM genes were knocked out (DKO cells) (right); cells were stained with anti-TACSTD2 (horizontal axis) and anti-EpCAM (vertical axis) antibodies. (D) Phase contrast image of DKO cells. Scale bar: 50 μm. (E) Results of immunostaining analysis for TACSTD2 and EpCAM in HCE-T-9 cells and DKO cells. Scale bar: 50 μm. (F) Results of Western blotting analysis for CLDN1, CLDN4, CLDN7, ZO-1, and GAPDH proteins in HCE-T-9 cells, TACSTD2-KO HCE-T-9 cells, and DKO cells. (G) Results of densitometric analysis of the Western blotting images. Chemiluminescent signals of indicated proteins were first normalized by those of GAPDH protein and further normalized to those of HCE-T-9 cells. Error bars: standard deviations of measurements. Asterisks indicate statistical significance among the three types of cells at P < 0.05 (1-way ANOVA). Daggers (†) indicate statistical significance between the indicated combinations at P < 0.017 (Student's t-test with Bonferroni correction). (H) Results of immunostaining analysis for CLDN1, CLDN4, CLDN7, and ZO-1 in HCE-T-9 cells and DKO cells. Scale bar: 50 μm. (I) Magnified image of CLDN7 immunostaining in DKO cells. Phalloidin (green) was used to indicate cell-cell borders. Scale bar: 10 μm. (J) Results of TER measurements for HCE-T-9 cells, TACSTD2-KO HCE-T-9 cells, and DKO cells. Error bars: standard deviations of measurements. Asterisks indicate statistical significance among the three types of cells at P < 0.05 (1-way ANOVA). Daggers (†) indicate statistical significance between the indicated combinations at P < 0.017 (Student's t-test with Bonferroni correction).

We successfully constructed TALEN plasmids for *EpCAM* (Fig. 4A). The cutting efficiency of the TALEN plasmids was significantly higher than negative control and even significantly higher than both the positive and super-positive controls, as confirmed by SSA assay (Fig. 4B). The TALEN plasmids were transfected into TACSTD2-KO HCE-T-9 cells. After 7 days, FACS analysis revealed that approximately 0.7% of TALEN-transfected TACSTD2-KO HCE-T-9 cells were devoid of *EpCAM* gene expression (Fig. 4C). These HCE-T cells, which did not express either *TACSTD2* or *EpCAM* (DKO cells) (Fig. 4D), were collected for subsequent analyses.

Immunostaining analysis confirmed that DKO cells did not express either *TACSTD2* or *EpCAM* gene (Fig. 4E). By Western blotting analysis, we found significantly decreased expression of CLDN1, CLDN4, and CLDN7 proteins in DKO cells (Fig. 4F, 4G). Notably, immunostaining analysis revealed that CLDN1 and CLDN7 proteins were localized in the cytoplasm, while CLDN4 and ZO-1 remained on the cell membrane (Fig. 4H, 4I); this is nearly identical to the subcellular localization pattern found in GDLD corneal epithelial cells.^18^ The TER of the DKO cells was significantly decreased, compared with that of HCE-T-9 cells, and was even significantly lower than that of TACSTD2-KO HCE-T-9 cells (Fig. 4J).

### Transduction of *TACSTD2* into DKO Cells

**Figure 5:**
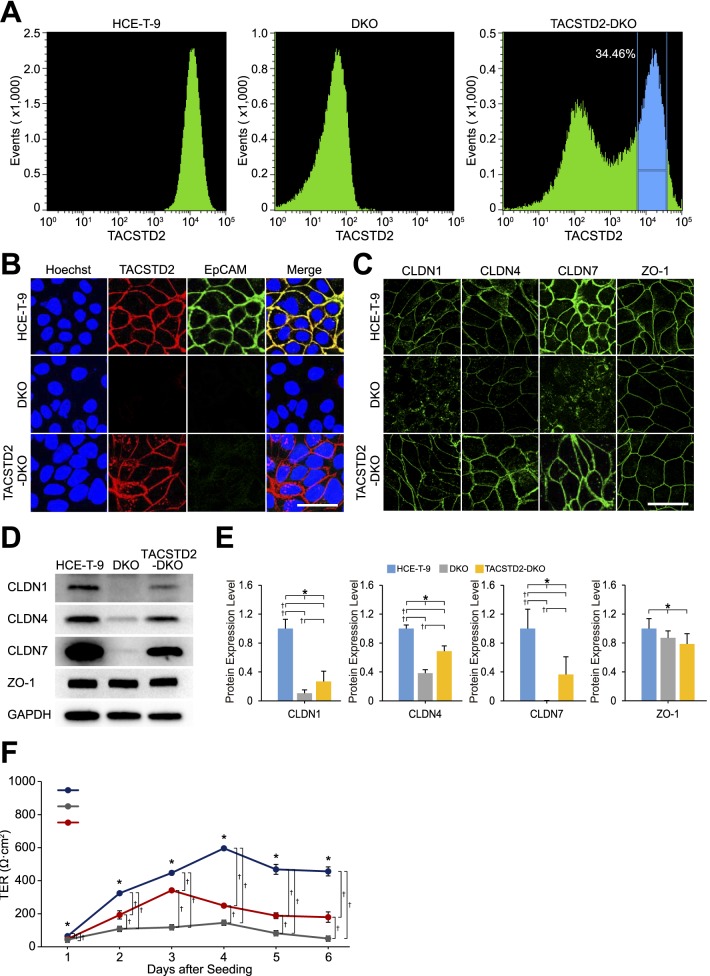
Transduction of TACSTD2 into DKO cells. (A) Results of FACS analysis for HCE-T-9 cells (left), DKO cells (middle), and DKO cells transduced with TACSTD2 (TACSTD2-DKO cells) (right); cells were stained with anti-TACSTD2 antibody. (B) Results of immunostaining analysis for TACSTD2 and EpCAM in HCE-T-9 cells, DKO cells, and TACSTD2-DKO cells. Scale bar: 50 μm. (C) Results of immunostaining analysis for CLDN1, CLDN4, CLDN7, and ZO-1 in HCE-T-9 cells, DKO cells, and TACSTD2-DKO cells. Scale bar: 50 μm. (D) Results of Western blotting analysis for CLDN1, CLDN4, CLDN7, ZO-1, and GAPDH proteins in HCE-T-9 cells, DKO cells, and TACSTD2-DKO cells. (E) Results of densitometric analysis of the Western blotting images. Chemiluminescent signals of indicated proteins were first normalized by those of GAPDH protein and further normalized to those of HCE-T-9 cells. Error bars: standard deviations of measurements. Asterisks indicate statistical significance among the three types of cells at P < 0.05 (1-way ANOVA). Daggers (†) indicate statistical significance between the indicated combinations at P < 0.017 (Student's t-test with Bonferroni correction). (F) Results of TER measurements in HCE-T-9 cells, DKO cells, and TACSTD2-DKO cells. Error bars: standard deviations of measurements. Asterisks indicate statistical significance among the three types of cells at P < 0.05 (1-way ANOVA). Daggers (†) indicate statistical significance between the indicated combinations at P < 0.017 (Student's t-test with Bonferroni correction).

*TACSTD2* was transduced into DKO cells to investigate whether exogenous provision of the gene could rescue the pathological phenotype of these cells. FACS was used to selectively obtain cells expressing *TACSTD2* gene at levels equal to those of HCE-T-9 cells (Fig. 5A, 5B). Immunostaining analysis of *TACSTD2* gene-transduced DKO cells (TACSTD2-DKO cells) revealed that CLDN1, CLDN4, and CLDN7 proteins were localized on the cell membrane (Fig. 5C). Western blotting analysis revealed that the expression levels of CLDN1, CLDN4, and CLDN7 proteins in TACSTD2-DKO cells were significantly increased compared with those in DKO cells, although they were still lower than those in HCE-T-9 cells (Fig. 5D, 5E). The TER of TACSTD2-DKO cells was significantly increased compared with DKO cells, although it was still lower than that of HCE-T-9 cells (Fig. 5F).

## Discussion

In the present study, we established a model cell line by knocking out *TACSTD2* and *EpCAM* genes in HCE-T cells. These cells exhibited a significant reduction in epithelial barrier function, decreased expression level, and altered subcellular localization from cell membrane to cytoplasm in CLDN1 and CLDN7 proteins. These characteristics are consistent with those of GDLD corneal epithelial cells. The established cell line, which appropriately reflects the pathological alterations in GDLD corneal epithelial cells, may serve as a good in vitro model of GDLD cornea.

HCE-T cells reportedly exhibit alterations in their genomic contents and may comprise multiple subclones with diverse cellular properties, despite their derivation from a single clone.^24^ This suggests that HCE-T cells may have lost their intrinsic cellular properties and do not constitute appropriate basal cells for the generation of GDLD model cells. Despite this concern, we ultimately selected HCE-T cells for several reasons. First, HCE-T cells have been utilized in numerous studies as a reliable in vitro model of corneal epithelial cells.^26,27^ Second, HCE-T cells express major components of tight junction proteins, including ZO-1, occludin, and CLDN.^28^ Third, HCE-T cells express the same set of CLDN proteins (CLDN1, 4, and 7) as in vivo corneal epithelial cells^29^; this suggests that HCE-T cells still maintain their original cellular properties, at least with respect to epithelial barrier function. Practically, the expression of CLDN1 and CLDN7 proteins was essential for our study because altered expression and subcellular localization of these proteins is the most characteristic pathophysiological change observed in GDLD cornea.^17^ The fact that some HCE-T subclones exhibited quite high epithelial barrier function was also the reason.

Kitazawa et al.^18^ previously established an in vitro disease model of GDLD by immortalization of corneal epithelial cells of GDLD patients (HCE-GDLD). These cells also reflected the pathological characteristics of GDLD corneal epithelial cells. However, compared to the use of the HCE-GDLD cells, there seems to be several advantages in our DKO cells. First, the culture process of our DKO cells involves a single step, while that of the HCE-GDLD cells is a two-step process. HCE-GDLD cells are first cultured in growth medium until cells reach confluence. Upon reaching confluence, the cells are cultured in a differentiation medium. This two-step culture process is complicated and may lead to undesired fluctuation in the data. Second, our DKO cells appear as a simple epithelium, whereas HCE-GDLD cells appear as a multilayered epithelium. It is easier to observe subcellular localization of tight junction-related proteins in a simple epithelium compared with a multilayered epithelium. Third, the growth medium for HCE-GDLD cells (CnT-Prime Epithelial Culture Medium) is a specialized serum-free medium and is quite expensive as compared with the culture medium used for our DKO cells (SHEM medium).

In the present study, we first knocked out the *TACSTD2* gene in HCE-T cells. The resulting TACSTD2-KO HCE-T cells exhibited reduced epithelial barrier function, which is consistent with the phenotype of GDLD corneal epithelial cells. However, CLDN7 protein remained localized on the cell membrane. Because altered subcellular localization of CLDN7 protein from cell membrane to cytoplasm seems to be one of the most characteristic pathological features of GDLD corneal epithelial cells, we suspected that TACSTD2-KO HCE-T cells did not adequately reflect the pathological phenotype of GDLD corneal epithelial cells.

*EpCAM* is the most paralogous gene of *TACSTD2*: these genes share 50% sequence similarity at the amino acid sequence level. Similar to TACSTD2, EpCAM has been reported to bind to CLDN1 and CLDN7, protecting them from lysosomal degradation.^25,30,31^ *EpCAM* mutant mice showed defective intestinal barrier function and died shortly after birth as a result of intestinal erosion.^32^ In the present study, EpCAM was expressed in HCE-T cells. Thus, we suspected that *EpCAM* compensated for lost *TACSTD2* gene function in TACSTD2-KO HCE-T cells.

The additional knockout of the *EpCAM* gene from TACSTD2-KO HCE-T cells resulted in further reduction in epithelial barrier function and dramatic changes in subcellular localization of CLDN1 and CLDN7 proteins, from cell membrane to cytoplasm. This indicates that *EpCAM* compensated for the lost TACSTD2 function in TACSTD2-KO HCE-T cells, as we had suspected. We also found that corneal epithelial cells did not express *EpCAM* in vivo. This is concordant with the fact that dysfunctional mutations of *TACSTD2* cause GDLD in humans. If *EpCAM* were expressed in corneal epithelial cells in vivo, it might compensate for lost *TACSTD2* gene function and prevent the appearance of clinical manifestations of GDLD.

The reason for *EpCAM* gene expression in HCE-T cells remains unknown. *EpCAM* is expressed in nonstratified epithelia, including simple epithelium of the gastrointestinal tract, ciliated pseudostratified epithelium of the airway, and transitional epithelium of the bladder.^33,34^ In contrast, *TACSTD2* is expressed in stratified epithelia, including corneal epithelium, conjunctival epithelium, esophageal epithelium, and skin epidermis.^17^ Considering their cellular origin, HCE-T cells are expected to express *TACSTD2*, but not *EpCAM*. HCE-T cells were reported to stratify in the original description of their establishment as a cell line.^35^ However, in our experience with HCE-T cells, they have never stratified and consistently appear as a simple epithelium. Therefore, we suspect that HCE-T cells had already lost the nature of stratified epithelium and gained that of simple epithelium, which might explain the expression of *EpCAM* in HCE-T cells. Alternatively, since many studies have demonstrated that the expression of *EpCAM* was closely correlated with carcinogenesis,^36^ the immortalization process of HCE-T cells might lead to *EpCAM* gene expression. Regarding the latter hypothesis, it is interesting that tumor protein p53, whose function is inhibited by simian virus 40 large T antigen,^37^ has been reported to suppress expression of the *EpCAM* gene through direct binding to the cis-regulatory element of *EpCAM* gene.^38^

In summary, we have established an HCE-T–based cell line by knocking out *TACSTD2* and *EpCAM* to generate an in vitro model of GDLD cornea. This cell line adequately reflected the pathological phenotypes of GDLD corneal epithelial cells. We expect that the cell line will be useful for further elucidation of the pathogenesis of GDLD cornea. We also expect that the cell line will be useful for the development of novel treatments for GDLD cornea, such as gene therapy or therapeutic small molecule compounds.
